# Molecular markers of response and toxicity to FOLFOX chemotherapy in metastatic colorectal cancer

**DOI:** 10.1038/sj.bjc.6605239

**Published:** 2009-08-11

**Authors:** W Chua, D Goldstein, C K Lee, H Dhillon, M Michael, P Mitchell, S J Clarke, B Iacopetta

**Affiliations:** 1 Department of Medical Oncology, Sydney Cancer Centre, Concord Repatriation General Hospital, Sydney, NSW, Australia; 2 Department of Medical Oncology, Prince of Wales Hospital, Concord, NSW, Australia; 3 NHMRC Clinical Trials Centre, Sydney, NSW, Australia; 4 Centre of Medical Psychology and Evidence-based Decision-Making, University of Sydney, NSW, Australia; 5 Division of Haematology and Medical Oncology, Peter MacCallum Cancer Centre, Melbourne, Victoria, Australia; 6 Department of Medical Oncology, Austin Hospital, Heidelberg, Victoria, Australia; 7 School of Surgery, University of Western Australia, Nedlands, Australia

**Keywords:** colorectal cancer, metastatic, chemotherapy, predictive factors

## Abstract

**Background:**

To investigate three genetic alterations (*TP53* mutation, *Kras* mutation and microsatellite instability (MSI)) and three polymorphisms (methylene tetrahydrofolate reductase (*MTHFR*) C677T, excision repair cross complementing group 1 (*ERCC1*)-118 and X-ray repair cross complementing group 1 (*XRCC1*)-399) for their ability to predict response, survival and toxicity to FOLFOX first line chemotherapy in the treatment of metastatic colorectal cancer (mCRC).

**Methods:**

Tumour tissues from 118 mCRC patients who underwent FOLFOX treatment from three successive phase II trials were evaluated for mutations in *TP53* (exons 5–8) and *Kras* (codons 12 and 13) and for MSI using PCR-based analysis. Genotyping for common single nucleotide polymorphisms in the *MTHFR* (codon 677), *ERCC1* (codon 118) and *XRCC1* (codon 399) genes was also carried out using PCR techniques. These genetic markers were correlated with clinical response, survival and toxicity to treatment.

**Results:**

Patients with the T allele of *ERCC1*-118 showed significantly worse progression-free survival in univariate analysis (HR=2.62; 95% CI=1.14–6.02; *P*=0.02). None of the genetic alterations or polymorphisms showed significant association with clinical response to FOLFOX. The *MTHFR*, *ERCC1* and *XRCC1* polymorphisms showed no associations with overall haematological, gastrointestinal or neurological toxicity to FOLFOX, although *MTHFR* 677 TT genotype patients showed a significantly higher incidence of grade 3 or 4 diarrhoea (26%) compared with CC or CT genotype patients (6%, *P*=0.02).

**Conclusions:**

The *ERCC1*-118 and *MTHFR* C677T polymorphisms were associated with progression and severe diarrhoea, respectively, after FOLFOX treatment in mCRC. Although our findings require confirmation in large prospective studies, they reinforce the concept that individual genetic variation may allow personalized selection of chemotherapy to optimize clinical outcomes.

There have been significant developments in chemotherapy regimes for the treatment of metastatic colorectal cancer (mCRC) over the past decade with the introduction of new cytotoxic drugs including oxaliplatin, irinotecan, capecitabine and biological agents. Oxaliplatin in combination with a fluoropyrimidine has become one of the most common first line chemotherapy regimens used for mCRC and its efficacy has been confirmed in this setting (Giachetti *et al*, 2000; de Gramont *et al*, 2000; Cassidy *et al*, 2004; Colucci *et al*, 2005). The rapidly evolving disciplines of molecular oncology and pharmacogenetics aim to correlate gene mutations and polymorphisms, respectively, with drug efficacy and toxicity. Such information might allow treatments to be tailored to suit individual patients, thus sparing them from unnecessary toxicity and expense while still achieving the best response. Numerous studies have investigated novel predictive factors in tumour tissue and blood that could allow such individualised therapy. To date, however, none of these markers has been introduced into routine clinical practice for the treatment of CRC, with the exception of recent and consistent evidence for *Kras* mutation being predictive for non-response to anti-EGFR treatment (Amado *et al*, 2008; Bokemeyer *et al*, 2008; Karapetis *et al*, 2008; de Roock *et al*, 2008; van Cutsem *et al*, 2008).

Because of its role in DNA repair, the *TP53* gene has been widely investigated as a possible predictor of response to chemotherapy. The results of a large international collaborative study indicate that wild-type *TP53* status is predictive of good response to 5-fluorouracil (5-FU)-based therapies in CRC (Russo *et al*, 2005). Mutation of the *Kras* oncogene has also been widely investigated as a prognostic factor in CRC (Andreyev *et al*, 1998); however, the predictive value of this marker for response to 5-FU is less well known. Another genetic alteration that has received considerable attention is the microsatellite instability (MSI) phenotype. Although some studies indicate that MSI is associated with poor response to 5-FU (Ribic *et al*, 2003), others suggest the contrary (Elsaleh *et al*, 2000; Kim *et al*, 2007). In addition to somatic genetic alterations found in tumour DNA, polymorphisms within the germline DNA may also have predictive value for response and toxicity to chemotherapy. This is because single nucleotide polymorphisms can alter enzyme activity and expression levels. One of the most well-studied polymorphisms in relation to the prediction of response and toxicity to 5-FU-based treatments is the C677T polymorphism in the methylene tetrahydrofolate reductase (*MTHFR*) gene (Etienne-Grimaldi *et al*, 2007). The mutant form of this gene is associated with lower *MTHFR* enzymatic activity leading to changes in the distribution of tissue folates. Similarly, tumour response to oxaliplatin may be influenced by polymorphisms in genes involved in the nucleotide excision repair pathway (Marsh and McLeod, 2004; Reed, 2005). These include polymorphisms in codon 118 (AAC to AAT) of the excision repair cross complementing group 1 (*ERCC1*) gene and codon 399 (CGG to CAG) of the X-ray repair cross complementing group 1 (*XRCC1*). Several groups have published on the predictive value of the *ERCC1*-118 polymorphism for response to oxaliplatin treatment in mCRC (Stoehlmacher *et al*, 2004; Viguier *et al*, 2005; Moreno *et al*, 2006; Ruzzo *et al*, 2007; Martinez-Balibrea *et al*, 2008; Pare *et al*, 2008), although the results have not been consistent. Less work has been published on the *XRCC1*-399 polymorphism and again the results have not been concordant (Stoehlmacher *et al*, 2004; Ruzzo *et al*, 2007; Pare *et al*, 2008).

The aim of this study was to evaluate three genetic alterations (*TP53* mutation, *Kras* mutation and MSI) and three polymorphisms (*MTHFR* C677T, *ERCC1*-118 and *XRCC1*-399) for their ability to predict response and toxicity to FOLFOX first line chemotherapy in the treatment of mCRC. Tissue samples were obtained from patients enrolled in three successive and prospective phase II trials investigating modifications of the FOLFOX4 regimen and the utility of gabapentin for reduction of oxaliplatin-based neuropathy (Goldstein *et al*, 2005; Michael *et al*, 2006; Mitchell *et al*, 2006).

## Materials and methods

The eligibility criteria for inclusion in the trials have been described earlier (Goldstein *et al*, 2005; Michael *et al*, 2006; Mitchell *et al*, 2006). Patients with a histologically confirmed diagnosis of advanced stage adenocarcinoma of the colon or rectum, who were chemotherapy naive were eligible to enter these trials. Patients were required to have measurable disease, adequate organ function, good performance status (ECOG performance status 0–2) and to have completed adjuvant treatment at least 6 months before entry into the trial. Patients were excluded if they had received earlier adjuvant chemotherapy with oxaliplatin. This substudy of molecular markers was performed with the approval of the individual institutional ethics committees where patients received treatment. A total of 134 patients were enrolled in the three trials and of these, primary tumour tissue samples were available for 118 (88%) patients.

### Molecular analyses

Formalin fixed and paraffin-embedded tumour tissue blocks of surgical resection or biopsy specimens were retrieved from pathology archives. After histological confirmation of tumour cell content, several 10-μm sections were cut and the DNA extracted for PCR amplification as described earlier (Soong and Iacopetta, 1997). Fluorescent single strand conformation polymorphism analysis (SSCP) was used to screen for mutations in exons 5–8 of *TP53* (Iacopetta *et al*, 2000a), codons 12 and 13 of *Kras* (Wang *et al*, 2003) and for MSI in the BAT-26 mononucleotide repeat (Iacopetta and Grieu, 2000b). Fluorescent SSCP was also used to determine the *MTHFR* C677T genotype (Grieu *et al*, 2004). Genotyping for the *ERCC1* codon 118 (C>T) and *XRCC*1 codon 399 (G>A) polymorphisms was carried out using the *Bsr*DI (Biolab, Australia) and *Msp*I (Promega, Australia) restriction enzymes and the PCR primers and conditions described earlier (Stoehlmacher *et al*, 2004).

### Chemotherapy response, toxicity and survival

Chemotherapy cycles were administered every 2 weeks until disease progression or the development of unacceptable toxicity. Radiological response was assessed as per the World Health Organisation Criteria (Miller *et al*, 1981). All toxicity was graded according to the National Cancer Institute Common Toxicity Criteria and toxicity assessments performed at day 1 of every cycle until the end of treatment. For this study, patients with complete or partial response were classified as responders, and patients with stable disease or progressive disease were classified as non-responders. Patients were also analysed according to disease stabilisation (complete response, partial response and stable disease) and progressive disease. Progression-free survival (PFS) was defined as the time from patient registration on clinical trial until the first documented tumour progression or death from any cause. Overall survival (OS) was defined as the time from patient registration to death from any cause.

### Statistical analysis

Fisher's exact tests were used to access association between genotypes and polymorphisms with response or toxicity outcome. Odds ratio and 95% confidence intervals (CIs) were reported on the basis of univariate logistic regression models. Time to event data were analysed using the Kaplan–Meier method. Hazard ratio (HR) and 95% CI were reported on the basis of univariate Cox proportion hazards regression analyses. No adjustment to the *P*-value was performed for multiple testing. Multivariate models were also constructed to evaluate the effect of genotypes and polymorphisms on PFS and OS after adjustment for other prognostic factors.

## Results

Clinical characteristics for the 118 patients evaluated in this study are shown in Table 1. Patients were predominantly male (68%) and the median age at diagnosis was 61 years (range, 31–75 years). A total of 102 patients (86%) had an ECOG performance status of 0 or 1. There was no evidence of heterogeneity in patient characteristics across the three trials. Information on clinical response to treatment was available for 106 patients, of whom 58 (55%) showed complete or partial response. The median PFS was 7.5 months (95% CI=6.0–8.6 months) and median OS was 16.3 months (95% CI=13.7–21.1 months). Major adverse events (grade 3 or 4 toxicity) were neutropenia (36% of patients), neurological toxicity (17%) and diarrhoea (9%).

Tumour samples for the 118 patients were analyzed at a single institution for the presence of mutations and for genotype status. No results for any marker were obtained for 1 patient, for MSI and *MTHFR* genotype in 2 patients, and for *TP53* mutation, *ERCC1* and *XRCC1* genotypes in 3 patients.

*TP53* mutation was found in 43 out of 115 (37%) cases, *Kras* mutation in 37 out of 117 (32%) cases and MSI+ in 2 out of 116 (2%) cases. *TP53* and *Kras* mutations showed no significant associations with clinical response to FOLFOX treatment. Only two cases showed MSI, of which one was associated with clinical response and the other was not. Genotype frequencies for *MTHFR* C677T were 37% (CC), 47% (CT) and 16% (TT), for *ERCC1*-118 they were 9% (CC), 56% (CT) and 35% (TT), whereas for *XRCC1*-399 they were 34% (GG), 53% (GA) and 13% (AA). No significant associations were seen between these polymorphisms and clinical response (Table 2) or disease stabilization (data not shown).

**Figure 1:**
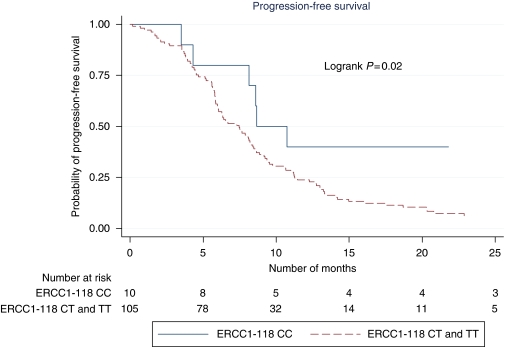
Progression-free survival and ERCC1-118 polymorphism.

The *ERCC1-118* polymorphism was significantly linked to PFS in univariate analysis (Table 3; Figure 1). Patients carrying a C/T or T/T genotype showed significantly worse PFS compared with those with the CC genotype (HR=2.62; 95% CI=1.14–6.02; *P*=0.02), but not for OS (*P*=0.2). The median PFS for patients with the *ERCC1-118* CC genotype was 8.7 months compared with 7.5 months for those with at least one T allele. In multivariate analysis, the association between *ERCC1-118* genotype and PFS approached statistical significance (*P*=0.07; Table 4). *TP53* mutation, *Kras* mutation, *MTHFR* genotype and *XRCC1* genotype were not associated with either PFS or OS (Table 3).

Somatic mutations are restricted to tumour tissue and were, therefore, not analyzed in relation to the prediction of systemic toxicity to treatment. The *MTHFR-C677T*, *ERCC1-118* and *XRCC1-399* polymorphisms showed no significant associations with overall haematological, gastrointestinal or neurological toxicity to treatment (Table 5). There were no associations when the results were analysed for toxicity after 3 or 6 months on treatment or the entire course of chemotherapy. Although only 11 patients experienced grade 3 or 4 diarrhoea, the *MTHFR* TT genotype was over-represented in this group (Table 6). The percentage of cases who suffered this severe toxicity was significantly higher for TT genotype patients (5 out of 19, 26%) than for CC or CT genotype patients (6 out of 97, 6%; *P*=0.02, Fisher's exact test).

## Discussion

The aims of this study were to investigate the associations of *TP53* mutation, *Kras* mutation and MSI and response to FOLFOX in mCRC, as well as the predictive values of polymorphisms in *MTHFR*, *ERCC1* and *XRCC1* for both response and toxicity to this treatment. These genetic markers were evaluated in 118 patients, of which clinical response data were available for 106 patients. The frequencies of *TP53* mutation (37%) and *Kras* mutation (32%) observed here are similar to those reported in other large studies of CRC (Andreyev *et al*, 1998; Russo *et al*, 2005). The very low frequency of MSI+ (2%) is in keeping with the low propensity for these tumours to metastasise (Jass, 2006) and prevented investigation of the predictive value of this molecular marker in the current study of mCRC. The distribution of ERCC1 and XRCC1 polymorphisms were similar to those described in other Caucasian populations (Ruzzo *et al*, 2008) with a slightly higher frequency of the *MTHFR 677* CC genotype in our population.

The *TP53* gene has important functions in DNA damage repair and apoptosis (Royds and Iacopetta, 2006). Its lack of association with response and survival to FOLFOX in this study (Tables 2 and 3) was therefore surprising and contrary to a large study reporting the impact of *TP53* on patients with Dukes' C tumours treated with 5-fluorouracil as adjuvant chemotherapy (Russo *et al*, 2005). The current finding that *Kras* mutation is not associated with response to FOLFOX supports an earlier study with 5-FU monotherapy (Etienne-Grimaldi *et al*, 2008). However, the strong predictive value of *Kras* mutation for response to anti-EGFR therapies has now been clearly established for mCRC (Amado *et al*, 2008; Bokemeyer *et al*, 2008; de Roock *et al*, 2008; Karapetis *et al*, 2008; van Cutsem *et al*, 2008). The MTHFR enzyme has a central function in regulating the pool of intracellular folates available for nucleic acid synthesis and DNA methylation. The common C677T polymorphism in *MTHFR* shows reduced enzyme activity that is hypothesized to increase intracellular folate concentrations and therefore increase sensitivity to fluoropyrimidines (Sohn *et al*, 2004). In support of this, human cancer cell lines with the *MTHFR* 677 T allele show greater sensitivity to 5-FU compared with those with the C allele (Sohn *et al*, 2004) and this was confirmed in a study of 98 CRC patients treated with 5-FU-based chemotherapy (Etienne *et al*, 2004). Similarly, patients with the *MTHFR* T allele are postulated to experience greater toxicity from fluoropyrimidine-based chemotherapy (Sharma *et al*, 2008).

The *MTHFR* C677T polymorphism was not associated with response to FOLFOX in this study. This result concurs with several other studies that used FOLFOX or FOLFIRI (Marcuello *et al*, 2006; Suh *et al*, 2006; Ruzzo *et al*, 2007) but not with two others that used 5-FU monotherapy and reported better response for patients with the TT genotype (Jakobsen *et al*, 2005; Etienne-Grimaldi *et al*, 2007). Thus, it appears that the type of chemotherapy regimen used could influence the predictive value observed for the *MTHFR* C677T polymorphism. A retrospective study reported the *MTHFR* A1298C polymorphism was associated with survival in 5-FU-treated mCRC, but only in female patients (Zhang *et al*, 2008). This polymorphism is in strong linkage disequilibrium with the C677T polymorphism; however, no gender difference in predictive value was observed here for the *MTHFR* C677T polymorphism.

Only a few studies have investigated *MTHFR* genotype as a predictor of toxicity to 5-FU-based chemotherapy and to our knowledge there have been no reports in relation to FOLFOX. The *MTHFR* C677T polymorphism was not associated with worse grade 3 or 4 haematological, gastrointestinal or neurological toxicity (Table 5). However, patients with the TT genotype suffered a significantly higher incidence of grades 3–4 diarrhoea (5 out of 19, 26%) compared with those with the CC or CT genotype (6 out of 97, 6%; Table 6). Interestingly, an earlier study with UFT/leucovorin found that 1 of 2 patients with the *MTHFR* TT genotype developed grade 3 diarrhoea at the first dose level (Veronese *et al*, 2004). Preliminary data published in abstract form suggest that 75% of patients receiving adjuvant 5-FU-based chemotherapy who had combined *MTHFR* 677 TT and 1298 AA genotypes predicted for severe grades 3–4 toxicities (Adamo *et al*, 2008). However, a large German study found that neither the *MTHFR* C677T nor the A1298C polymorphisms were associated with toxicity to 5-FU in cancer patients (Schwab *et al*, 2008), supporting earlier observations in patients treated with 5-FU monotherapy (Cohen *et al*, 2003), FOLFOX (Ruzzo *et al*, 2007) or FOLFIRI (Ruzzo *et al*, 2008). Capitain *et al* (2008) reported the A1298C polymorphism, but not C677T, was predictive of toxicity to 5-FU (Capitain *et al*, 2008). Clearly, more work in larger cohorts that includes analysis of both *MTHFR* polymorphisms is required to determine whether these genetic variants are associated with 5-FU toxicity to, particularly for diarrhoea. The collection of additional information on blood or tissue folate status would also be very useful in helping to clarify the predictive significance of *MTHFR* polymorphisms.

*ERCC1* and *XRCC1* are both involved in the repair of DNA damage and hence functional variants of these genes are candidate predictive markers for response to oxaliplatin. Unfortunately, results to date on the predictive value of the *ERCC1*-118 polymorphism for response to oxaliplatin-based chemotherapy have been inconsistent. Several groups have reported that mCRC patients with the *ERCC1*-118 TT or CT genotype had better tumour response or survival compared with CC patients (Viguier *et al*, 2005; Moreno *et al*, 2006; Martinez-Balibrea *et al*, 2008; Pare *et al*, 2008). However, two groups reported the T allele was associated with worse survival (Park *et al*, 2002; Ruzzo *et al*, 2007). Stoehlmacher *et al* (2004) reported a two-fold increase risk of dying for patients with the C/T or T/T genotype compared with those with a C/C genotype (Stoehlmacher *et al*, 2004). Consistent with the results by Ruzzo *et al* (2007) and Stoehlmacher *et al* (2004), patients in this study with an *ERCC1*-118 CT or TT genotype had a 2.6-fold greater risk of progression with FOLFOX chemotherapy compared with those with the CC genotype. In multivariate analysis, this result approached statistical significance (HR=2.16; 95% CI=0.94–4.97; *P*=0.07), with organ involvement and baseline neutrophil count being significant for PFS (Table 3). Our multivariate analysis replicated a recent report highlighting the value of clinical factors in predicting risk (Sanoff *et al*, 2008). *ERCC1* protein expression was not assessed in this; however, two earlier studies reported an association between low expression of ERCC1 (mRNA and protein) and improved overall survival in CRC (Shirota *et al*, 2001; Kim *et al*, 2009).

Park *et al* (2002) earlier showed a trend towards higher mRNA levels with increasing numbers of *ERCC1*-118 T alleles (Park *et al*, 2002). ERCC1 is important for the removal of DNA adducts caused by platinum compounds and hence the increased gene expression in CT and TT individuals may lead to treatment resistance. This could explain the worse PFS seen for these individuals in this study of FOLFOX treatment (Table 3). However, contrary results have been found in studies of ovarian cell lines, where the *ERCC1* codon 118 C–T substitution was associated with reduced levels of ERCC1 mRNA and protein expression (Yu *et al*, 2000). The functional consequences of the silent *ERCC1*-118 polymorphism, therefore, remain unclear and may vary according to tissue type. The contrasting results from clinical studies of this *ERCC-1* polymorphism may be due to small sample sizes and type I (false positive) rates, with more definitive results likely to be achieved through meta-analysis (Pajak *et al*, 2000).

Fewer studies have been carried out on polymorphisms in *XRCC1* as an important factor for response to oxaliplatin-based chemotherapy. An initial report on the *XRCC1*-399 polymorphism suggested an association with response (Stoehlmacher *et al*, 2001); however, subsequent studies including one by the same group failed to confirm this finding (Stoehlmacher *et al*, 2004; Ruzzo *et al*, 2007; Pare *et al*, 2008). This study also failed to confirm an association between *XRCC1*-399 polymorphism and response or survival to FOLFOX (Tables 2 and 3). The lack of association with overall toxicity (Table 5) also suggests that *ERCC1* and *XRCC1* are not involved in adverse reactions to FOLFOX treatment, consistent with results from other studies (Ruzzo *et al*, 2007). In our study, there was also no correlation between neurotoxicity and response, PFS and OS. These data, together with our findings on the value of MTHFR as a predictor of toxicity but not efficacy, reinforce the complexity of the impact of these genetic changes in predicting tailored chemotherapy treatments.

This biological substudy was conducted on three successive phase II trials with prospectively collected information on response, toxicity and survival outcomes. The large number of samples available for analysis added to the strength of the study. Genetic polymorphisms were analysed in tumour tissue rather than germline DNA. Although the majority of pharmacogenetic studies have been performed in germline DNA, almost complete concordance between germline and somatic DNA has been found in terms of variants within pharmacogenetic genes (Marsh *et al*, 2005; McWhinney and Mcleod, 2009).

In conclusion, this study found significant associations between the *ERCC1*-118 CC genotype and improved PFS and between the *MTHFR* 677 TT genotype and grade 3 or 4 diarrhoea after FOLFOX treatment. The present findings indicate that *TP53* mutation, *Kras* mutation and MSI are unlikely to be clinically useful molecular markers for the prediction of response to FOLFOX chemotherapy in mCRC. Similarly, the *MTHFR* C677T, *ERCC1*-118 and *XRCC1-*399 polymorphisms were not associated with clinical response to FOLFOX. Our observations on the association of *ERCC1*-118 and *MTHFR* C677T polymorphisms for response and toxicity, respectively, to FOLFOX in mCRC require confirmation in large prospective studies. Emerging information in this area based on prospective trials should lead to clinically useful information becoming available in the future.

## Figures and Tables

**Table 1:** Baseline characteristics of mCRC patients

**Characteristic**	***n***	**%**
Total number	118	100
		
*Gender*		
Male	80	68
Female	38	32
		
*Primary tumour*		
Colon	82	70
Rectum	36	30
		
*Ethnic origin*		
Caucasian	114	96
Asian	2	2
Others	2	2
		
*ECOG performance status*		
0	50	42
1	52	44
2	14	12
		
*Prior adjuvant chemotherapy*		
Yes	83	70
No	35	30
		
Carcinoembryonic antigen (*μ*g l^-1^)		17
Median (range)		(0–12, 900)

mCRC=metastatic colorectal cancer.

**Table 2:** Clinical response according to molecular features

	**Patients**		**Clinical response** a		
**Marker**	***n***	**%**	**Odds ratio**	**95% CI**	***P***
*TP53 mutation*					
Absent	72	63	1.00		
Present	43	37	1.67	0.74–3.73	0.2
					
*Kras mutation*					
Absent	80	68	1.00		
Present	37	32	1.21	0.54–2.73	0.6
					
*MSI*					
Absent	114	98	1.00		
Present	2	2	0.79	0.05–12.97	0.9
					
*MTHFR 677*					
CC	43	37	1.00		
CT	54	47	0.42	0.18–0.99	0.05
TT	19	16	0.67	0.20–2.17	0.5
CT and TT	73	63	0.47	0.21–1.06	0.07
					
*ERCC1-118*					
CC	10	9	1.00		
CT	64	56	0.35	0.06–1.86	0.2
TT	41	35	0.51	0.09–2.88	0.4
CT and TT	105	91	0.40	0.08–2.10	0.3
					
*XRCC1-399*					
GG	39	34	1.00		
AG	61	53	1.00	0.43–2.34	1.0
AA	15	13	1.28	0.35–4.68	0.7
AG and AA	76	66	1.05	0.46–2.37	0.9

a Complete response/partial response *vs* stable disease/progressive disease.

**Table 3:** Univariate Cox proportional hazards regression for progression-free survival (PFS) and overall survival (OS)

	**Patients**		**PFS**			**OS**		
**Marker**	***n***	**%**	**HR**	**95% CI**	***P***	**HR**	**95% CI**	***P***
*TP53 mutation*								
Absent	72	63	1.00			1.00		
Present	43	37	0.85	0.58–1.26	0.4	0.88	0.56–1.37	0.6
								
*Kras mutation*								
Absent	80	68	1.00			1.00		
Present	37	32	0.91	0.60–1.36	0.6	0.76	0.48–1.21	0.2
								
*MSI*								
Absent	114	98	1.00			1.00		
Present	2	2	0.36	0.05–2.62	0.3	0.40	0.06–2.92	0.4
								
*MTHFR 677*								
CC	43	37	1.00			1.00		
CT	54	47	0.81	0.53–1.24	0.3	1.10	0.68–1.76	0.7
TT	19	16	1.05	0.61–1.81	0.9	1.35	0.75–2.45	0.3
CT and TT	73	63	0.87	0.59–1.29	0.5	1.16	0.75–1.81	0.5
								
*ERCC1-118*								
CC	10	9	1.00			1.00		
CT	64	56	2.68	1.15–6.23	0.02	1.88	0.75–4.71	0.2
TT	41	35	2.54	1.07–6.04	0.04	1.55	0.60–4.00	0.4
CT and TT	105	91	2.62	1.14–6.02	0.02	1.74	0.70–4.30	0.2
								
*XRCC1-399*								
GG	39	34	1.00			1.00		
AG	61	53	0.57	0.28–1.19	0.1	0.92	0.58–1.45	0.7
AA	15	13	1.01	0.39–2.60	1.0	0.52	0.24–1.14	0.1
AG and AA	76	66	0.66	0.34–1.28	0.2	0.83	0.53–1.29	0.4

**Table 4:** Multivariable analysis of clinical and molecular factors and PFS

**Variable**	**Hazard ratio**	**95% CI**	***P***
*Number of organs involved*			
1	1.00		
>1	1.62	1.21–2.15	0.001
			
*Absolute neutrophil count at baseline*			
< ULN^a^	1.00		
⩾ULN	1.12	1.03–1.21	0.009
			
*ERCC1-118*			
CC	1.00		
CT and TT	2.16	0.94–4.97	0.07

a The upper limit of normal (ULN) for neutrophils was 7 × 10^9^ per litre.

**Table 5:** Associations between polymorphisms and overall haematological, gastrointestinal and neurological toxicity

	**Haematological**					**Gastrointestinal**					**Neurological**				
	**0–2**		**3–4**			**0–2**		**3–4**			**0–2**		**3–4**		
**Genotype**	***n***	**%**	***n***	**%**	***P***	***n***	**%**	***n***	**%**	***P***	***n***	**%**	***n***	**%**	***P***
*MTHFR 677*															
CC	24	56	19	44		37	86	6	14		33	77	10	23	
CT	40	74	14	26		50	93	4	7		49	91	5	9	
TT	12	63	7	37	0.17	14	74	5	26	0.10	14	74	5	26	0.10
															
*ERCC1–118*															
CC	7	70	3	30		10	100	0	0		9	90	1	10	
CT	43	67	21	33		56	88	8	12		55	86	9	14	
TT	25	61	16	39	0.77	35	85	6	15	0.44	33	81	8	19	0.66
															
*XRCC1–399*															
GG	9	60	6	40		13	87	2	13		15	100	0	0	
AG	41	67	20	33		53	87	8	13		49	80	12	20	
AA	25	64	14	36	0.86	35	90	4	10	0.90	33	85	6	15	0.17

**Table 6:** Incidence of diarrhoea according to *MTHFR* C677T genotype

**Genotype**	**Grades 0–2, *n* (%)**	**Grades 3–4, *n* (%)**	***P***
*MTHFR* 677			
CC	40 (38)	3 (27)	
CT	51 (49)	3 (27)	
TT	14 (13)	5 (45)	0.02^a^

a Fisher's exact test (TT *vs* CT/CT genotype).
